# Cardiovascular Benefits of *Salvia miltiorrhiza* (Danshen) Supplementation: Bioactive Constituents, Mechanisms, Clinical Evidence and Implications for Nutritional Care

**DOI:** 10.1002/fsn3.71755

**Published:** 2026-04-26

**Authors:** Akash Vikal, Rashmi Maurya, Preeti Patel, Balak Das Kurmi, William N. Setzer, Javad Sharifi‐Rad, Daniela Calina

**Affiliations:** ^1^ Sahu Onkar Saran School of Pharmacy, Faculty of Pharmacy IFTM University Moradabad India; ^2^ Department of Pharmaceutics ISF College of Pharmacy Moga Punjab India; ^3^ Department of Pharmaceutics ISF College of Pharmacy Moga India; ^4^ Aromatic Plant Research Center Suite Lehi USA; ^5^ Department of Chemistry University of Alabama in Huntsville Huntsville Alabama USA; ^6^ Universidad Espíritu Santo Samborondón Ecuador; ^7^ Department of Medicine, College of Medicine Korea University Seoul Republic of Korea; ^8^ Centro de Estudios Tecnológicos y Universitarios del Golfo Veracruz Mexico; ^9^ Department of Clinical Pharmacy University of Medicine and Pharmacy of Craiova Craiova Romania

**Keywords:** cardiovascular therapy, Danshen, drug bioavailability, endothelial function, natural products, oxidative stress

## Abstract

Cardiovascular diseases (CVDs) remain the leading cause of mortality worldwide, creating interest in adjunctive strategies that complement standard care. *Salvia miltiorrhiza* (Danshen), a traditional Chinese medicinal herb rich in tanshinones and salvianolic acids, has been investigated for multi‐target cardiovascular effects. This narrative review summarizes key phytochemical classes, preclinical mechanisms, human clinical findings, and formulation approaches aimed at improving bioavailability. Preclinical studies consistently report antioxidative, anti‐inflammatory, vasodilatory, antithrombotic, and anti‐fibrotic activities, involving pathways such as endothelial nitric oxide signaling and modulation of NF‐κB/MAPK‐related inflammation. Clinical studies have reported potential benefits in conditions such as coronary artery disease and myocardial ischemia; however, the overall evidence is limited by heterogeneity in preparations and dosing, variable product composition, small sample sizes, and a shortage of rigorously designed trials. Practical translation is further constrained by poor solubility and rapid metabolism of key constituents, though delivery platforms (e.g., liposomes, nanoparticles, and self‐microemulsifying systems) may enhance exposure in experimental settings. Overall, Danshen remains a plausible adjunct candidate, but standardization and high‐quality randomized clinical trials are needed to establish efficacy, safety, and interaction risk with conventional cardiovascular therapies.

## Graphical abstract

*Salvia miltiorrhiza* (Danshen) contains bioactive tanshinones, including tanshinone IIA, cryptotanshinone, and tanshinone I, that exert cardioprotective effects through coordinated modulation of vascular endothelial growth factor receptor (VEGFR), transforming growth factor beta 1 (TGF‐β1), transforming growth factor beta receptor (TGF‐βR), toll‐like receptor 4 (TLR4), nuclear factor kappa B (NF‐κB), NOD‐like receptor family pyrin domain containing 3 (NLRP3), Janus kinase 2 (JAK‐2), signal transducer and activator of transcription 3 (STAT‐3), and Smad signaling, thereby promoting angiogenesis and attenuating cardiac fibrosis, inflammation and apoptosis.

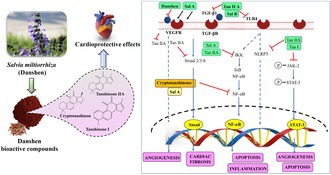

**Abbreviations**
CHD: coronary heart disease
CVDs: cardiovascular diseases
CYP: cytochrome P450
ECM: extracellular matrix
MI: myocardial Infarction
MMP: matrix metalloproteinase
PEG: polyethylene glycol
P‐gp: P‐glycoprotein
ROS: reactive oxygen species
Sal‐A: salvianolic cid A
Sal‐B: salvianolic cid B
SLNs: solid lipid nanoparticles
SM:
*Salvia miltiorrhiza*

SMEDDS: self‐microemulsifying drug delivery system
S‐SMEDDS: solid self‐microemulsifying drug delivery system
TCM: traditional chinese medicine

## 1 Introduction

Cardiovascular diseases (CVDs) are the main cause of mortality in the world, responsible for nearly 17.9 million fatalities annually. This category of conditions affects the heart and blood vessels, encompassing ailments such as coronary artery disease, cerebrovascular disorders, and rheumatic heart disease. Alarmingly, over 80% of deaths linked to CVDs stem from heart attacks and strokes, with one‐third occurring prematurely in individuals younger than 70 years old (Di Cesare et al. 2024). Despite the availability of various conventional treatments such as medications, lifestyle modifications, surgical interventions, and rehabilitative therapies, the global burden of CVD continues to rise (Ullah et al. 2023). While these conventional approaches help manage symptoms and reduce complications, they often have significant drawbacks (Kaiser et al. 2020). Medications, including blood thinners, statins, beta‐blockers, and nitrates, though effective, are related to adverse effects such as uncontrolled bleeding, liver dysfunction, muscle pain, fatigue, and dizziness. Similarly, surgical interventions like stent placement, coronary artery bypass grafting, and pacemaker implantation are invasive, carry potential complications, and do not always provide a lasting solution (Netala et al. 2024). Moreover, lifestyle modifications, despite being a cornerstone of prevention, demand long‐term commitment and may not be sufficient for individuals with advanced CVD. Given these limitations, there is a growing need for alternative and complementary therapies to provide a more comprehensive and sustainable approach to cardiovascular health (Ghodeshwar et al. 2023; Hossain et al. 2025).

Traditional Chinese Medicine (TCM) has received growing recognition as a possible supplement or substitute for traditional CVD therapies (Wang et al. 2024). Among the various herbal remedies in TCM, Danshen ( *Salvia miltiorrhiza* Bunge) stands out for its well‐documented cardioprotective effects. This medicinal herb has been used for centuries to improve blood circulation, decrease oxidative stress, and mitigate the progression of atherosclerosis and hypertension (Wang et al. 2017; Lin and Hsieh 2010). Studies suggest that Danshen possesses anti‐inflammatory, vasodilatory, and anticoagulant properties, making it a favorable candidate for CVD management with potentially fewer side effects than standard pharmaceuticals (Li et al. 2023). Given the urgent need for more effective and well‐tolerated treatments for CVD, this review delves into the potential of Danshen in cardiovascular therapy, exploring its mechanisms of action, clinical efficacy, and possible integration into modern treatment strategies (Dai et al. 2024). By bridging traditional knowledge with contemporary research, this review aims to comprehensively understand Danshen's role in CVD management and its potential to complement or enhance current therapeutic approaches (Netala et al. 2024). Although several narrative reviews, systematic reviews, and meta‐analyses have summarized Danshen's cardiovascular effects, they often focus on either pharmacology/mechanisms or pooled clinical outcomes. In contrast, this review provides an updated translational synthesis that (i) links key constituents and quality/standardization issues to mechanistic pathways, (ii) interprets human evidence in the context of recent systematic reviews and meta‐analyses, and (iii) integrates formulation/advanced delivery strategies and practical safety considerations (including herb–drug interaction risk) to better inform clinical and nutritional applicability.

## 2 Literature Search and Study Selection

This review employed a structured approach to examine the pharmacological properties of Danshen in relation to cardiovascular health, drawing on both experimental and clinical evidence. The methodology included an extensive literature search, eligibility criteria, data extraction, and qualitative synthesis. A comprehensive search was conducted in PubMed, Scopus, Web of Science, and Google Scholar to identify relevant articles published up to 2025. The search used keywords including “Danshen,” “ *Salvia miltiorrhiza* ,” “cardiovascular disease,” “myocardial infarction,” “hypertension,” “atherosclerosis,” “stroke,” and “clinical trials,” and Boolean operators (AND/OR) were applied to refine results. Studies were included if they were peer‐reviewed and reported preclinical or clinical findings on Danshen and cardiovascular outcomes. Studies examining pharmacological mechanisms, therapeutic effects, and safety of Danshen extracts or isolated bioactive compounds were prioritized. Only English‐language articles were included. Non‐peer‐reviewed sources (e.g., commentaries, editorials, opinion pieces) were excluded. Studies with insufficient methodological detail, those not focused on cardiovascular relevance, and duplicate or inconclusive reports were omitted. Data were extracted on study design, model (in vitro, in vivo, or clinical), sample size, intervention details (dose and formulation), outcomes, pharmacokinetic parameters, and main conclusions. Discrepancies in interpretation were resolved through discussion among reviewers. Findings were synthesized qualitatively and grouped by experimental model and clinical relevance, with emphasis on mechanisms, therapeutic potential, and safety considerations.

## 3 Phytochemistry, Bioactive Constituents, and Standardization of *Salvia miltiorrhiza*

*Salvia miltiorrhiza* (SM), usually known as Danshen, is a perennial herb from the Lamiaceae (mint) family, native to China and Japan, and widely cultivated or introduced in Korea and Vietnam. It thrives in temperate climates, preferring well‐drained soils and mountainous regions, and is typically harvested in autumn when its bioactive compounds reach peak concentration (Xing et al. 2024). SM is widely used in traditional Chinese medicine, with the roots serving as the principal medicinal part for cardiovascular indications. These effects are mainly attributed to two major classes of bioactives: lipophilic diterpenoid tanshinones and hydrophilic phenolic acids, particularly salvianolic acids.

**FIGURE 1:**
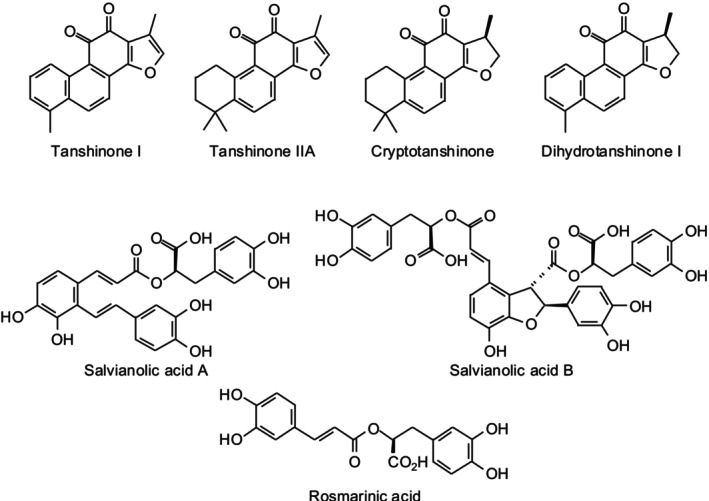
Chemical structures of major bioactive constituents from *Salvia miltiorrhiza* .

These compounds exhibit significant cardioprotective, antioxidant, anti‐inflammatory, and anti‐thrombotic properties, making Danshen a promising alternative or complementary therapy for CVDs (Su et al. 2015). Tanshinones, tanshinone I, tanshinone IIA, cryptotanshinone, and dihydrotanshinone I are the most lipophilic constituents associated with the anti‐thrombotic, anti‐atherosclerotic, and vasodilating activities of the herb (Figure 1) (Wang et al. 2020).

Tanshinone IIA, in particular, has been thoroughly researched for its capacity to prevent myocardial ischemia–reperfusion injury, inhibit vascular smooth muscle proliferation, and reduce oxidative stress (Wang et al. 2024). Cryptotanshinone enhances endothelial nitric oxide (NO) production, promoting vasodilation, while dihydrotanshinone I modulates inflammatory pathways and protects cardiac cells from oxidative damage (Zhou et al. 2006). The mechanism of action of tanshinones involves scavenging reactive oxygen species (ROS), inhibiting platelet aggregation, reducing inflammation through NF‐κB and TNF‐α pathways, and regulating calcium channels to prevent arrhythmias (Li et al. 2018).

Salvianolic acids, such as salvianolic acid A, salvianolic acid B, and rosmarinic acid, contribute to Danshen's antioxidant, anti‐thrombotic, and vasoprotective properties (He et al. 2023). Salvianolic acid B, the most abundant and well‐studied phenolic acid, enhances endothelial nitric oxide synthase (eNOS) activity, prevents LDL oxidation, and inhibits platelet aggregation, thus lowering the likelihood of atherosclerosis and high blood pressure. These compounds also protect endothelial cells by reducing oxidative stress and improving vascular elasticity, which helps maintain proper blood flow and prevents clot formation (Ho and Hong 2011; Wu et al. 2020).

The extraction and standardization of Danshen bioactives are fundamental for ensuring high yield, purity, and pharmacological efficacy. Conventional extraction methods include solvent extraction using ethanol, methanol, or water, which is widely used but has limitations in selectivity and efficiency (Zhang, Zhao, et al. 2023). Cutting‐edge methods, including ultrasound‐enhanced extraction (UAE), supercritical fluid extraction (SFE), and microwave‐assisted extraction (MAE), have been devised to enhance the extraction effectiveness and bioavailability of bioactive compounds (Shen et al. 2023; Bhadange et al. 2024). Chromatographic techniques like high‐performance liquid chromatography (HPLC), ultra‐performance liquid chromatography (UPLC), and liquid chromatography‐mass spectrometry (LC–MS) are used for the quantification and standardization of key bioactive markers, primarily tanshinone IIA and salvianolic acid B (Vanitha Madhuri et al. 2024). Despite its therapeutic potential, the pharmacokinetics and bioavailability of Danshen compounds pose challenges due to poor solubility and rapid metabolism (Mahalakshmi et al. 2021). Tanshinones, being highly lipophilic, have low water solubility, which limits their absorption. Strategies such as liposomal formulations, nanoemulsions, and solid dispersion systems have been explored to improve their bioavailability (Wang et al. 2021). Although more water‐soluble, salvianolic acids undergo rapid metabolism in the liver via glucuronidation and sulfation, leading to reduced systemic availability (Li et al. 2006). Novel drug delivery systems (DDS), including polymeric nanoparticles and sustained‐release formulations, are being investigated to enhance their therapeutic potential (Begines et al. 2020). The phytochemical complexity of SM highlights its potential as a multi‐targeted cardiovascular therapy. By modulating oxidative stress, improving endothelial function, and preventing thrombosis, Danshen offers a promising complementary approach to conventional CVD treatments (Gao and Hou 2023). Future research focusing on enhancing bioavailability, optimizing extraction techniques, and understanding molecular mechanisms will be fundamental for integrating Danshen into modern cardiovascular pharmacotherapy (Liu et al. 2022).

## 4 Mechanistic Insights: Preclinical Evidence and Molecular Pharmacology

Danshen has been widely studied for its cardiovascular protective effects. Its bioactive compounds, including tanshinones, salvianolic acids, and diterpenoids, exert multiple pharmacological actions through various molecular mechanisms (Li et al. 2018). Below is a detailed exploration of Danshen's role in endothelial function, nitric oxide modulation, anti‐inflammatory and antioxidant properties, platelet aggregation, lipid metabolism, and cardiac remodeling (Table 1).

**TABLE 1:** Molecular mechanisms underlying the cardiovascular effects of key Danshen bioactive compounds.

Mechanism	Compound	Molecular targets	Pathways modulated	Outcomes	References
Vascular inflammation suppression	Salvianolic Acid A	↓ ICAM‐1 ↓ VCAM‐1 ↓ MCP‐1 ↓ IL‐1β, ↓ COX2 ↓ iNOS	↓ NF‐κB ↓ JAK/STAT ↓ IKKβ ↓ PDGFRβ/ERK ↑ cAMP/PKA/CREB	Inhibits endothelial inflammation and proliferation; antithrombotic effect	Teng et al. (2016), Chen et al. (2017)
NO production and lipid regulation	Salvianolic Acid B	↑ eNOS, ↓ NF‐κB, ↑ LDL receptors ↓ CD36 ↓ CXCR4	↑ PI3K/Akt, ↑ AMPK ↑ PPARγ/LXRα/ABCA1 ↑ Nrf2/HO‐1	↑ NO synthesis; Improves lipid metabolism ↑endothelial protection	Zhao et al. (2023), Franceschelli et al. (2023)
Anti‐fibrotic and antithrombotic action	Tanshinone IIA	↓ TGF‐β1 ↓ Smad ↓ COX‐1 ↓ TXA2 ↓ ICAM‐1 ↓ ET‐1	↓ TGF‐β1/Smad, ↓ MAPK, ↓ JAK/STAT, ↑ PI3K/Akt, ↑ Nrf2/HO‐1	Reduces fibrosis, platelet aggregation, and inflammation; enhances vasodilation	Shi et al. (2020), Tran et al. (2022), Yang et al. (2018)
Antioxidant	Salvianolic Acid B	↓ ROS ↑ SOD ↑ CAT ↓ oxLDL uptake	↑ Nrf2/HO‐1	Boosts antioxidant defenses and endothelial resistance to oxidative damage	He et al. (2023), Sun et al. (2009)
Mitochondrial function restoration	Cryptotanshinone	↓ MMP‐9 ↓ LOX1 ↑ ATP synthase	↓ NF‐κB, ↓ AP‐1 ↓ mitochondrial ROS	Reduces VSMC proliferation, Improves energy balance Suppresses inflammation	Jin et al. (2009)

Abbreviations: ↑, upregulation or activation; ↓, downregulation or inhibition; ABCA1, ATP‐binding cassette transporter A1; Akt, protein kinase B; AMPK, AMP‐activated protein kinase; AP‐1, activator protein 1; ATP, adenosine triphosphate; CAT, catalase; CD36, cluster of differentiation 36; COX‐1/COX2, cyclooxygenase‐1/2; CREB, cAMP response element‐binding protein; CXCR4, C‐X‐C chemokine receptor type 4; eNOS, endothelial nitric oxide synthase; ERK, extracellular signal‐regulated kinase; ET‐1, endothelin‐1; HO‐1, heme oxygenase‐1; ICAM‐1, intercellular adhesion molecule‐1; IKKβ, inhibitor of nuclear factor kappa‐B kinase subunit beta; IL‐1β, interleukin‐1 beta; iNOS, inducible nitric oxide synthase; JAK/STAT, Janus kinase/signal transducer and activator of transcription; LDL, low‐density lipoprotein; LOX1, lectin‐like oxidized LDL receptor‐1; LXRα, liver X receptor alpha; MCP‐1, monocyte chemoattractant protein‐1; MMP‐9, matrix metallopeptidase 9; NF‐κB, nuclear factor kappa‐light‐chain‐enhancer of activated B cells; NO, nitric oxide; Nrf2, nuclear factor erythroid 2‐related factor 2; PDGFRβ, platelet‐derived growth factor receptor beta; PI3K, phosphoinositide 3‐kinase; PKA, protein kinase A; PPARγ, peroxisome proliferator‐activated receptor gamma; ROS, reactive oxygen species; SOD, superoxide dismutase; Smad, mothers against decapentaplegic homolog; TXA2, thromboxane A2; VCAM‐1, vascular cell adhesion molecule‐1; VSMC, vascular smooth muscle cell.

### 4.1 Endothelial Function and Nitric Oxide Modulation

Endothelial dysfunction is a major contributor to CVDs, characterized by reduced nitric oxide (NO) bioavailability and increased oxidative stress (Ray et al. 2023). Danshen enhances endothelial function through multiple mechanisms. Salvianolic acid B stimulates endothelial nitric oxide synthase (eNOS) activity via the PI3K/Akt pathway, promoting NO synthesis and improving vascular relaxation (Cheng et al. 2021). Additionally, its polyphenolic compounds inhibit NADPH oxidase while enhancing superoxide dismutase (SOD) activity, reducing ROS that degrade NO. Furthermore, tanshinone IIA facilitates vascular regeneration by stimulating the proliferation and migration of endothelial progenitor cells (Qin et al. 2023). Through these actions, Danshen helps restore vascular homeostasis, prevent endothelial dysfunction, and lower hypertension risk (Bernard and Thannickal 2020).

### 4.2 Anti‐Inflammatory and Antioxidant Properties

Chronic inflammation and oxidative stress are key drivers of cardiovascular disease (CVD) progression, and Danshen exerts significant anti‐inflammatory and antioxidant effects through multiple pathways (Yan et al. 2023). Salvianolic acids help suppress the expression of pro‐inflammatory cytokines such as TNF‐α, IL‐6, and IL‐1β by blocking NF‐κB activation, thereby reducing inflammation (He et al. 2023). Additionally, tanshinones and salvianolic acids act as free radical scavengers, enhancing the activity of endogenous antioxidants like glutathione peroxidase (GSH‐Px) and catalase (CAT) to mitigate oxidative damage (Sun et al. 2009). Danshen also modulates inflammatory signaling by inhibiting the MAPK and JAK/STAT pathways, which reduces inflammatory cell infiltration and prevents tissue damage. Through these mechanisms, Danshen contributes to cardioprotection, reduces vascular inflammation, and prevents atherosclerosis (Guo et al. 2024).

### 4.3 Platelet Aggregation and Thrombotic Pathways

Danshen is well known for its antithrombotic effects, which play a fundamental role in preventing stroke, myocardial infarction, and other thrombotic events. It inhibits platelet aggregation through salvianolic acid B and tanshinone IIA, which block ADP, thrombin, and collagen‐induced platelet activation by reducing intracellular calcium mobilization (Neves et al. 2024). Additionally, Danshen helps suppress fibrin clot formation by lowering the expression of fibrinogen and von Willebrand factor, thereby preventing thrombus development. It also downregulates thromboxane A2 (TXA2) synthesis, a potent vasoconstrictor and pro‐thrombotic agent, by inhibiting cyclooxygenase‐1 (COX‐1) (Kubatka et al. 2022). These combined mechanisms make Danshen a natural antiplatelet and anticoagulant agent, offering significant benefits in the prevention of cardiovascular complications (Yuan et al. 2024).

### 4.4 Lipid Metabolism and Atherogenesis

Dysregulated lipid metabolism is a key factor in atherosclerosis, leading to the buildup of plaques in arteries and increasing the risk of heart disease. Danshen helps regulate lipid levels through multiple mechanisms (Linton et al. 2019). Salvianolic acids enhance LDL receptor expression and activate AMPK, promoting lipid breakdown and reducing fat accumulation, which helps lower LDL cholesterol and triglycerides (Son et al. 2024). Additionally, Danshen compounds increase HDL cholesterol by upregulating ApoA1 and PPAR‐α, improving lipid transport and enhancing cholesterol efflux (Soltani et al. 2021). Moreover, Danshen prevents foam cell formation by inhibiting oxidized LDL (ox‐LDL) uptake, stopping macrophages from turning into foam cells—a fundamental step in atherosclerosis development. These actions collectively help reduce plaque formation, improve lipid profiles, and lower cardiovascular risk (Yang et al. 2018).

### 4.5 Cardiac Remodeling and Fibrosis

Cardiac remodeling, often triggered by hypertension, myocardial infarction, or heart failure, involves fibrosis, hypertrophy, and ventricular dysfunction (Azevedo et al. 2016). Danshen mitigates these effects through several mechanisms. Tanshinone IIA inhibits cardiac fibroblast activation by blocking the TGF‐β1/Smad signaling pathway, reducing collagen deposition and myocardial fibrosis (Shi et al. 2020). Additionally, salvianolic acids protect against myocardial apoptosis by activating the Nrf2/HO‐1 pathway, which lowers oxidative stress‐induced cardiomyocyte death (Ji et al. 2020). Danshen also improves mitochondrial function by enhancing ATP production and reducing mitochondrial ROS generation, preventing energy depletion in heart cells. Through these mechanisms, Danshen helps preserve cardiac structure and function, reducing the risk of heart failure progression (Pan et al. 2025; Chiu et al. 2012).

### 4.6 Compound‐Specific Mechanisms: Tanshinones and Salvianolic Acids in Cardiovascular Protection

**FIGURE 2:**
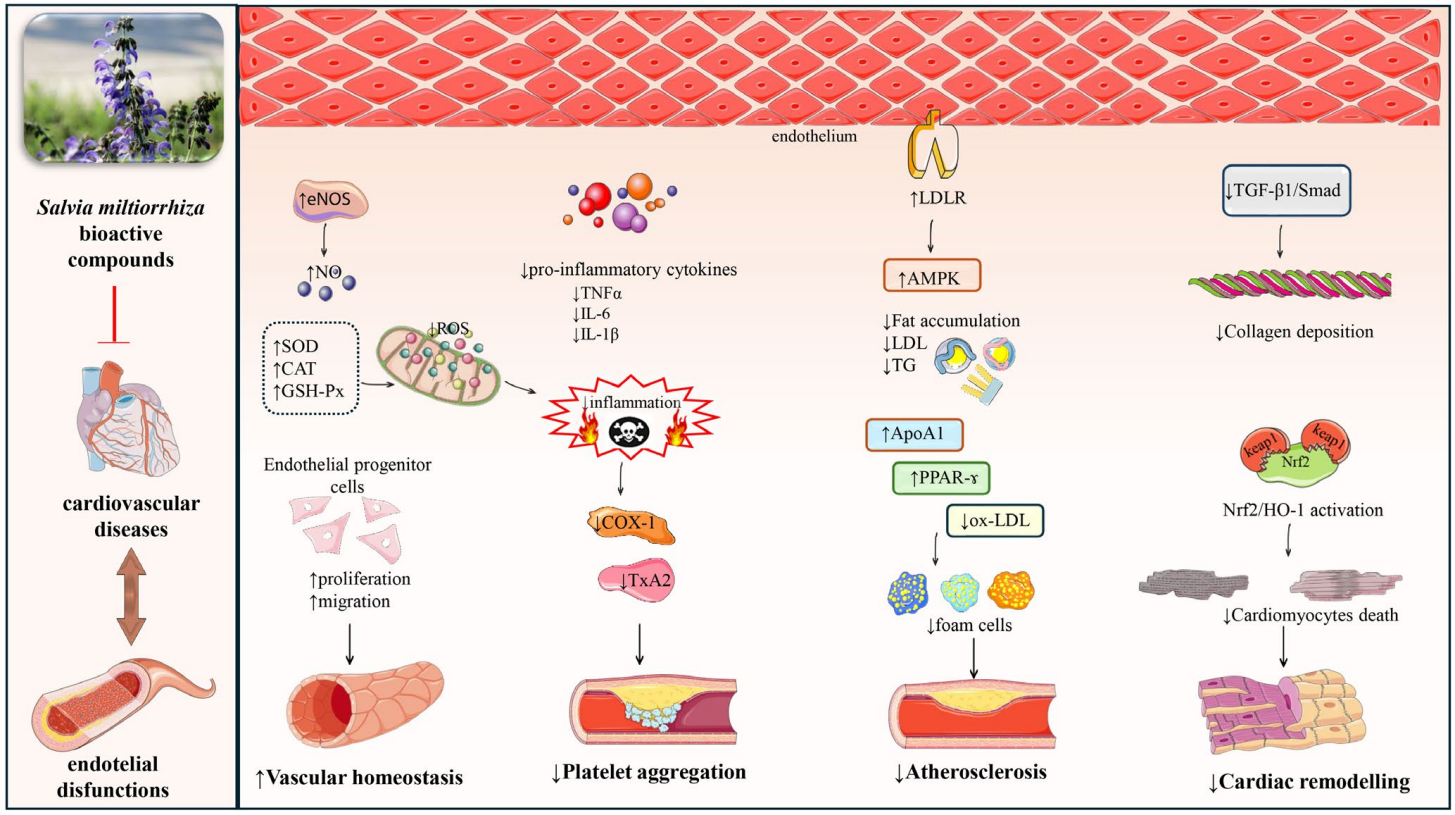
Cardioprotective mechanisms of *Salvia miltiorrhiza* bioactive compounds against cardiovascular diseases. Bioactive compounds such as tanshinones and salvianolic acids enhance endothelial function by upregulating eNOS and increasing nitric oxide (NO) production, while reducing reactive oxygen species (ROS) via antioxidant enzymes (SOD, CAT, GSH‐Px). Anti‐inflammatory effects are mediated by suppression of pro‐inflammatory cytokines (TNF‐α, IL‐6, IL‐1β), leading to inhibition of COX‐1 and thromboxane A2 (TxA2), ultimately reducing platelet aggregation. Lipid metabolism is modulated by upregulating LDL receptors (LDLR), ApoA1, and PPAR‐γ, as well as activating AMPK, which together reduce fat accumulation, LDL, and foam cell formation, contributing to the prevention of atherosclerosis. Anti‐fibrotic and cardioprotective effects are achieved through inhibition of the TGF‐β1/Smad pathway and activation of Nrf2/HO‐1, which reduce collagen deposition and cardiomyocyte death, thereby limiting cardiac remodeling. ↑, increase; ↓, decrease; AMPK, AMP‐activated protein kinase; ApoA1, apolipoprotein A1; CAT, catalase; COX‐1, cyclooxygenase‐1; CSE, cystathionine γ‐lyase; eNOS, endothelial nitric oxide synthase; FTH1, ferritin heavy chain 1; GSH‐Px, glutathione peroxidase; IL‐1β, interleukin‐1 beta; IL‐6, interleukin‐6; LDL, low‐density lipoprotein; LDLR, low‐density lipoprotein receptor; NO, nitric oxide; NQO‐1, NAD(P)H quinone dehydrogenase 1; Nrf2, nuclear factor erythroid 2–related factor 2; ox‐LDL, oxidized low‐density lipoprotein; PPAR‐γ, peroxisome proliferator‐activated receptor gamma; ROS, reactive oxygen species; SOD, superoxide dismutase; TGF‐β1, transforming growth factor‐beta 1; TG, triglycerides; TNF‐α, tumor necrosis factor‐alpha; TxA2, thromboxane A2.

Tanshinone IIA exerts its cardiovascular protective effects through multiple mechanisms (Figure 2). It protects endothelial cells by reducing oxidative stress‐induced injury via Nrf2 activation and inhibits inflammatory responses by suppressing TNF‐α (Tumor Necrosis Factor‐alpha), ICAM‐1 (Intercellular Adhesion Molecule‐1), VCAM‐1 (Vascular Cell Adhesion Molecule‐1), MCP‐1 (Monocyte Chemoattractant Protein‐1), E‐selectin (Endothelial Selectin), IL‐8 (Interleukin‐8), and IL‐1β (Interleukin‐1 β), thereby preventing monocyte adhesion (Yang et al. 2023). It also promotes vasodilation by enhancing NO bioavailability through eNOS activation/phosphorylation and by reducing endothelin‐1 (ET‐1) levels (Tran et al. 2022). Tanshinone IIA may reduce endothelial nitric oxide synthase (eNOS) uncoupling by restoring the tetrahydrobiopterin/dihydrobiopterin (BH4/BH2) balance and upregulating key regulators of BH4 homeostasis—GTP cyclohydrolase I (GTPCH1) and dihydrofolate reductase (DHFR)—together with heat shock protein 90 (HSP90). In vascular smooth muscle cells (VSMCs), it suppresses extracellular signal–regulated kinase (ERK) and 3‐phosphoinositide‐dependent protein kinase‐1 (PDK1) signaling and activates large‐conductance Ca^2+^‐activated K^+^ channels (BKCa), AMP‐activated protein kinase (AMPK), and nuclear factor erythroid 2–related factor 2 (Nrf2), which may help limit vascular remodeling and plaque instability (Ren et al. 2015). In macrophages, it exhibits anti‐atherogenic effects by blocking scavenger receptor‐mediated oxidized LDL (oxLDL) uptake, reducing foam cell formation, and promoting cholesterol efflux via ABCA1 and ABCG1 through the Nrf2/HO‐1 pathway. Moreover, it inhibits platelet aggregation and activation, contributing to its overall atheroprotective effects (Zhang, Wu, et al. 2023). Cryptotanshinone, another active compound of Danshen, shares similar protective effects by reducing endothelial inflammation, inhibiting LOX1‐mediated pro‐inflammatory signaling, and suppressing monocyte adhesion. It also prevents VSMC proliferation and migration by inhibiting MMP‐9 expression through the NF‐κB and AP‐1 pathways, further supporting its role in reducing atherosclerosis progression (Jin et al. 2009). Additionally, salvianolic acid A (Sal‐A) enhances endothelial function by increasing NO bioavailability through MKP‐3 inhibition, improving endothelial barrier function, and acting as an ET1 type A receptor (ETAR) antagonist to reduce vascular remodeling. It inhibits NF‐κB by targeting IKKβ, suppressing pro‐inflammatory mediators (COX2, iNOS, TNFα, IL‐6), and preventing Ang II‐induced endothelial proliferation by blocking ROS generation, Src, and Akt phosphorylation (Teng et al. 2016). Sal‐A also represses endothelial‐to‐mesenchymal transition (EndMT) via Nrf2 activation and Smad modulation while exhibiting anti‐thrombotic effects by inhibiting platelet aggregation through cAMP/PKA/CREB and PDGFRβ/ERK signaling (Chen et al. 2017). Salvianolic acid B (Sal‐B) improves endothelial function by decreasing TNFα‐induced adhesion molecule expression (ICAM‐1, VCAM‐1) via NF‐κB and AP1 inhibition, activates Nrf2/HO‐1 to prevent LDL oxidation and oxLDL‐induced endothelial injury, and inhibits VSMC proliferation and migration by blocking CXCR4 and activating Nrf2 (Zhao et al. 2023). Additionally, Sal‐B reduces foam cell formation by suppressing CD36‐mediated oxLDL uptake and enhancing cholesterol efflux through the PPARγ/LXRα/ABCA1 pathway (Franceschelli et al. 2023). The major bioactive constituents and their mechanisms are summarized in Table 1.

Representative preclinical models demonstrating cardioprotective effects are summarized in Table 2.

**TABLE 2:** Summary of preclinical models demonstrating cardioprotective effects of Danshen compounds.

Model	Tested compound	Dose/Concentration	Primary endpoint	Major findings	References
In vitro H9c2 cardiomyocytes	Salvianolic Acid B	10–50 μM	Oxidative stress, apoptosis	Reduced ROS, increased cell survival	Wang et al. (2018)
In vitro Human umbilical vein endothelial cells (HUVECs)	Tanshinone IIA	5–20 μM	Endothelial dysfunction	Enhanced NO production, reduced inflammation	Yang et al. (2023)
In vivo Rat model of myocardial infarction	Danshen extract	100 mg/kg (oral)	Infarct size, cardiac function	Reduced infarct size, improved ejection fraction	Wang et al. (2020)
In vivo Mouse model of atherosclerosis	Salvianolic Acid A	50 mg/kg (oral)	Atherosclerotic plaque formation	Decreased lipid accumulation, reduced inflammation	Xie et al. (2023)
In vivo Rat model of hypertension	Cryptotanshinone	20 mg/kg (intraperitoneal)	Blood pressure regulation	Lowered systolic BP, improved endothelial function	Li et al. (2020)

## 5 Clinical Studies

Danshen has been evaluated in human studies for cardiovascular indications (Table 3); however, the clinical evidence is heterogeneous in intervention type (extracts, multi‐herb formulations, injections), standardization, dosing, populations, and endpoints. Many trials have small sample sizes, short follow‐up, and incomplete reporting of randomization/blinding, which limits statistical power and confidence in effect estimates. Mechanistic findings from in vitro and animal studies should not be extrapolated to humans unless supported by clinical endpoints or relevant biomarkers. As this is a narrative review and the studies are not sufficiently comparable, no meta‐analysis was conducted; findings are summarized descriptively.

**TABLE 3:** Summary of clinical studies.

Model/Population	Dose/Form	Outcomes	References
Human adults > 40 years (with peripheral arterial disease)	Danshen Gegen Capsule (3 capsules (500 mg per capsule) twice daily for 24 weeks)	The study assesses changes in walking distance, quality of life (EuroQol‐5D), and functional status (Walking Impairment Questionnaire) over 24 weeks. It also evaluates cardiovascular event rates, ankle‐brachial index before and after exercise, and arterial stiffness (brachial‐to‐ankle pulse wave velocity) to measure vascular health improvements	NCT02380794 (2015) https://clinicaltrials.gov/study/NCT02380794
Human (with hypertension)	Danshen & Gegen 2 g, 1 g	Reduced hypertension symptoms in 1‐year observation	NCT01033630 (2009)
Human adults (with unstable angina)	Gualou Xiebai Banxia Decoction & Danshen Decoction	The study measures myocardial enzymes, blood lipids, and treadmill test results at baseline and four weeks, with myocardial infarction or heart failure incidence tracked over six months	NCT03179514 https://cdek.pharmacy.purdue.edu/trial/NCT03179514/
Human adults (with coronary heart disease, primary hypertension)	Danshen dripping pill		NCT01825759 https://cdek.pharmacy.purdue.edu/trial/NCT01825759/

## 6 Translational Cardiovascular Applications of *Salvia miltiorrhiza* : Integrative Evidence From Mechanisms to Clinical Validation

### 6.1 Therapeutic Perspectives

The bioactive compounds of Danshen are being considerably investigated for their cardiovascular benefits, with growing evidence supporting its use from molecular mechanisms to clinical applications. Its bioactive compounds, including tanshinones and salvianolic acids, exhibit diverse pharmacological effects such as vasodilation, anti‐thrombosis, anti‐inflammatory activity, and myocardial protection (Li et al. 2018). These properties have been explored in preclinical models and human trials, offering promising insights into their therapeutic potential. However, challenges such as standardization, safety concerns, and herb‐drug interactions must be carefully considered to ensure its effective integration into cardiovascular treatment strategies (Wang et al. 2023). This section provides a comprehensive review of mechanistic actions of Danshen, experimental validation, clinical evidence, safety considerations, and future research directions.

The cardiovascular benefits of SM are primarily attributed to its bioactive constituents, notably tanshinones and salvianolic acids, which exhibit a range of pharmacological activities (Jung et al. 2020). One of its key effects is antioxidative, as Tanshinone IIA has been shown to activate the Nrf2 pathway, enhancing the expression of antioxidative proteins such as NQO‐1 while promoting iron homeostasis through ferritin heavy chain 1 (FTH1). This mechanism plays a fundamental role in mitigating oxidative stress‐induced endothelial damage, a significant contributor to atherosclerosis development (He et al. 2021). Additionally, Danshen demonstrates strong anti‐inflammatory properties, with salvianolic acids reducing leukocyte‐endothelial adherence and inhibiting the expression of metalloproteinases in aortic smooth muscle cells, thereby decreasing vascular inflammation and remodeling. The herb also contributes to vasodilation by stimulating the production of endogenous hydrogen sulfide (H_2_S), a potent vasodilator, through the activation of the cystathionine γ‐lyase (CSE) pathway, which enhances blood vessel relaxation and helps regulate blood pressure (Ho and Hong 2011). Furthermore, its anti‐fibrotic properties have been linked to the ability of tanshinones to inhibit myocardial fibrosis by modulating signaling pathways such as PI3K/Akt, ultimately contributing to the preservation of cardiac function. Together, these mechanisms highlight the therapeutic potential of Danshen in cardiovascular protection (Jung et al. 2020).

Animal and in vitro studies provide fundamental evidence supporting the cardiovascular benefits of Danshen. Research has demonstrated its potential in models of atherosclerosis, myocardial infarction, and hypertension (Wang et al. 2017). Additionally, the treatment has been found to ameliorate ultrastructural changes in the thoracic aorta's adventitial fibroblasts, suggesting vascular protective properties (Liu et al. 2016). In atherosclerosis models, preclinical studies indicate that tanshinones and salvianolic acids help inhibit lipid accumulation and reduce plaque formation, likely due to their strong antioxidative and anti‐inflammatory properties (Xu et al. 2024). Furthermore, in myocardial infarction models, tanshinone IIA has demonstrated the ability to reduce infarct size and enhance cardiac function post‐myocardial infarction by modulating apoptotic pathways and boosting antioxidative defenses (Fang et al. 2021). These findings collectively support the potential of SM as a therapeutic agent for cardiovascular diseases.

Clinical investigations have highlighted the therapeutic potential of SM in various cardiovascular conditions (Lyu et al. 2021). In patients with coronary artery disease (CAD), clinical trials suggest that Danshen preparations can alleviate angina symptoms and enhance myocardial perfusion, effects largely attributed to the vasodilatory and anti‐thrombotic properties of its bioactive compounds (Dai et al. 2024). Additionally, research indicates that Danshen may be beneficial for individuals with heart failure by improving cardiac output and reducing symptoms, likely due to its positive inotropic and anti‐fibrotic effects. Furthermore, evidence supports its neuroprotective role in ischemic stroke, with studies suggesting that its antioxidative and anti‐inflammatory properties help mitigate ischemia–reperfusion injury, reducing brain damage and improving recovery outcomes (Lin and Hsieh 2010).

The bioavailability of SM is significantly restricted by poor solubility, extensive first‐pass metabolism, and rapid clearance, which hinder its therapeutic potential. The major bioactive components, including lipophilic tanshinones (tanshinone I, tanshinone IIA, cryptotanshinone, and dihydrotanshinone I) and hydrophilic salvianolic acids (Sal‐A and Sal‐B), face absorption challenges due to their chemical properties (Kim et al. 2008). Tanshinones have extremely low water solubility, leading to poor gastrointestinal absorption, while salvianolic acids, despite their hydrophilicity, exhibit rapid metabolism and a short elimination half‐life. Additionally, these compounds are substrates for P‐glycoprotein (P‐gp), which pumps them out of intestinal cells, further reducing systemic absorption (Xing et al. 2017). Extensive metabolism by cytochrome P450 (CYP) enzymes also contributes to their low bioavailability.

To overcome these challenges, various strategies have been explored, including nanoformulations such as liposomes, solid lipid nanoparticles (SLNs), and polymeric nanoparticles, which enhance solubility and protect against metabolism (Zhang, Dong, et al. 2021). Co‐administration with bioenhancers like piperine and polyethylene glycol (PEG) helps inhibit P‐gp efflux, improving intestinal absorption. Structural modifications and prodrug approaches, such as magnesium lithospermate B (a modified form of Sal‐B), have shown improved pharmacokinetics and therapeutic effects (Nguyen et al. 2021). Cyclodextrin inclusion complexes enhance solubility by forming stable drug complexes, while self‐microemulsifying DDSs (SMEDDS) improve oral absorption by facilitating dispersion in gastrointestinal fluids (Salawi 2022). Additionally, alternative delivery methods like transdermal patches and injectable formulations bypass first‐pass metabolism, ensuring higher systemic exposure. Despite these challenges, advancements in novel DDS continue to enhance the bioavailability of SM, making it a promising therapeutic agent for cardiovascular and inflammatory diseases (Ezike et al. 2023).

Besides this, Bi et al. formulated a solid self‐microemulsifying DDS (S‐SMEDDS) to improve the oral bioavailability of both fat‐soluble compounds (e.g., tanshinone IIA, tanshinone I, cryptotanshinone, dihydrotanshinone I) and water‐soluble compounds (e.g., danshensu, salvianolic acid B) of SM. The S‐SMEDDS formulation was refined through solubility analysis and phase diagram development to achieve excellent self‐emulsification properties; stability, optimal droplet size, polydispersity index, and zeta potential were ensured. The formulation was fabricated by adding a liposoluble extract into an aqueous solution containing a hydrophilic polymer and subjecting it to freeze‐drying. In vitro release experiments demonstrated that 60%–80% of each active is released in 20 min. In vivo rat bioavailability tests verified that the oral absorption of these constituents improved considerably with S‐SMEDDS compared to the drug suspension. The research concludes that S‐SMEDDS is a viable method for the delivery of SM extract and other multi‐component drugs, efficiently improving dissolution rates and oral absorption of compounds with different polarities (Bi et al. 2016).

A similar conclusion was reached by Pan et al., who studied the pharmacokinetics of cryptotanshinone, a key lipophilic constituent of SM, using a hydroxypropyl‐β‐cyclodextrin inclusion complex in dogs and rats. Given the low oral bioavailability of cryptotanshinone, the study aimed to enhance its absorption and systemic exposure. The inclusion complex improved bioavailability, which was 6.9% ± 1.9% in rats (60 mg/kg) and 11.1% ± 1.8% in dogs (53.4 mg/kg), compared to its conventional formulation. The drug exhibited slow absorption and dose‐proportional pharmacokinetics, with a half‐life of 5.3–7.4 h in rats and 6.0–10.0 h in dogs (Pan et al. 2008). These findings align with other studies on SM bioavailability enhancement strategies, such as the S‐SMEDDS developed by Bi et al., which improved the dissolution and absorption of both lipophilic (e.g., cryptotanshinone, tanshinone IIA) and hydrophilic (e.g., salvianolic acid B, danshensu) components. The pharmacokinetic improvements observed with cyclodextrin inclusion further highlight the importance of advanced delivery systems in enhancing the therapeutic efficacy of SM‐derived bioactives (Bi et al. 2016).

### 6.2 Safety, Toxicity, and Herb‐Drug Interactions

While Danshen is generally regarded as safe, certain considerations must be considered. Adverse effects are rare but can include mild gastrointestinal discomfort and allergic reactions in some individuals. One of the primary concerns is its potential for herb‐drug interactions, particularly with anticoagulant medications like warfarin (Zhou et al. 2005).

This interaction is largely attributed to the inhibition of cytochrome P450 enzymes, specifically CYP1A2 and CYP3A4, by tanshinones, which may alter drug metabolism and efficacy. As a result, careful monitoring is necessary when Danshen is used alongside medications processed by these enzymes (Qiu et al. 2010). From a clinical perspective, the main safety concern is bleeding risk when Danshen is used with antithrombotic therapy, so medication reconciliation and risk stratification are advisable in routine practice. Additionally, although the herb is well‐tolerated at recommended dosages, excessive intake may lead to toxicity, reinforcing the importance of adhering to appropriate dosing guidelines to minimize potential risks (Zhang, Luo, et al. 2021).

Extensive experimental and clinical research has demonstrated that Danshen, in both its crude form and various preparations (such as Danshen injection, Danshen dripping pill, Danhong injection, and Danshen‐Gegen decoction), provides significant cardioprotective benefits during pathological conditions like myocardial ischemia, myocardial infarction, and reperfusion injury (Jia et al. 2011). Key bioactive components of Danshen, including the lipophilic tanshinone IIa and cryptotanshinone, as well as the hydrophilic Danshensu, Sal‐A, and Sal‐B, exhibit potent cardioprotective effects. These compounds primarily protect the heart from acute ischemic injury through their antioxidant, anti‐inflammatory, and anti‐apoptotic properties (Wei et al. 2023). Additionally, some components contribute to mitigating pathological cardiac remodeling, highlighting their potential as therapeutic agents for chronic heart diseases such as heart failure (Fang et al. 2023). In 2023, Ma et al. explored the mechanism of SM and tanshinone IIA (Tan IIA) in treating atherosclerosis through network pharmacology and molecular biology experiments. They elucidated 35 significant overlapping targets between SM and coronary artery disease (CAD) and built a protein–protein interaction (PPI) network. GO and KEGG analyses demonstrated that SM reduces CAD mainly by anti‐inflammatory pathways targeting TNF, COX‐2 (PTGS2), MMP9, and NF‐κB. Molecular docking validated Tan IIA as the major active compound, inhibiting vascular inflammation and plaque formation through the COX‐2/TNF‐α/NF‐κB pathway. In vivo and in vitro experiments proved that SM and Tan IIA inhibited lipid deposition and inflammatory cytokine expression in ApoE−/− mice and ox‐LDL‐cultured HUVECs. Considering the drawbacks of traditional CAD therapy, their research validates SM and Tan IIA as novel plant‐derived therapeutics for the control of atherosclerosis (Ma et al. 2023).

Apart from this, Chang et al. examined SM effects on oxidative stress in CVDs related to aging via a systematic review of the literature. They observed that SM is an antioxidant, antiapoptotic, and anti‐inflammatory but also angiogenic and cardioprotective. SM suppresses ROS generation by blocking oxidases, inhibiting oxidative modification of low‐density lipoproteins, and reducing mitochondrial oxidative stress. It increases antioxidant enzyme function, such as catalase, MnSOD, glutathione peroxidase, and coupled eNOS. SM also decreases ischemia/reperfusion injury, suppresses cardiac fibrosis following myocardial infarction, maintains cardiac function in coronary artery disease, and sustains blood–brain barrier integrity and neural stem cell regeneration following stroke. These data indicate that SM may represent a novel therapeutic agent for CVDs, although well‐conducted clinical trials are essential to confirm its effectiveness (Chang et al. 2016). Furthermore, Wu et al. reviewed the cardiovascular protective effects of salvianolic acids A (SalA) and B (SalB), the primary water‐soluble bioactive compounds extracted from SM. These compounds have demonstrated potent antioxidant, anti‐inflammatory, and antifibrotic characteristics, rendering them suitable contenders for cardiovascular disease treatment. Comprehensive in vivo and in vitro research indicates that SalA and SalB alleviate oxidative stress, platelet clumping, blood clotting, thrombosis, endothelial dysfunction, and vascular inflammation by acting on various vascular cell types. As polyphenolic compounds, they possess direct ROS scavenging abilities, but more quantitative methods, such as electron spin resonance spin trapping, are needed to precisely measure these effects in pharmacokinetic studies. Additionally, improving formulation techniques to enhance their bioavailability and stability remains fundamental. The broad cardiovascular benefits of SalA and SalB provide a strong scientific rationale for their continued exploration as small‐molecule drug candidates for cardiovascular disease treatment (Wu et al. 2020).

Additionally, Zhang et al. investigated the additive effects of *Huang Qi* (HQ, *Astragalus mongholicus* ) and *Dan Shen* (DS, *Salvia miltiorrhiza* ) in treating coronary heart disease (CHD). Using network pharmacology and a myocardial infarction (MI) rat model, they identified 170 shared and specific genes targeted by HQ and DS. The combination therapy significantly improved cardiac function, reduced myocardial infarction size and fibrosis, regulated lipid metabolism, and maintained circulatory homeostasis more effectively than either herb alone. HQ specifically alleviated cardiac remodeling by reducing ventricular dilation and myocardial hypertrophy, while DS had a distinct advantage in regulating hypercoagulability and hemodynamics. Given the limitations of conventional CHD treatments such as aspirin and statins, HQ and DS offer a promising alternative with fewer side effects. Their combined effects on lipid metabolism, oxidative stress, inflammation, and fibrosis highlight their potential as complementary therapies for preventing heart failure post‐MI (Zhang, Wang, et al. 2021).

Furthermore, Wu et al. evaluated the antihypertensive effects of SABP, a combination of hydrophilic active metabolites from SM, in spontaneously hypertensive rats (SHRs). SABP was administered intraperitoneally, with perindopril as a control, and its impact on oxidative stress, inflammation, and vascular remodeling was assessed through histochemical staining, Western blot, and electron microscopy. Additionally, adventitial fibroblasts were isolated to evaluate their proliferation, migration, and transformation in response to SABP. Results showed that SABP significantly reduced systolic blood pressure, suppressed oxidative stress and inflammation via the ROS/TLR4/NF‐κB pathway, and inhibited vascular fibrosis and remodeling in SHRs. It also prevented fibroblast activation and myofibroblast differentiation through the TGFβ/Smad3 pathway. These outcomes highlight the adventitia's role in vascular dysfunction and suggest SABP as a promising alternative antihypertensive therapy. Future research will focus on its effects on peripheral arteries to further elucidate its therapeutic potential (Wu et al. 2023).

Additionally, Xia et al. investigated whether antioxidant therapy with SM extract prevents the postoperative increase of endothelin‐1 in children with congenital heart defects undergoing cardiopulmonary bypass. Twenty children with pulmonary hypertension were randomized into a placebo control group (A) or a treatment group (B) receiving SM (200 mg/kg intravenously). Blood samples were collected at multiple time points before, during, and after surgery to measure malondialdehyde (MDA), creatine kinase‐MB (CK‐MB), thromboxane B2, and prostacyclin metabolites. Results showed that MDA significantly increased in the placebo group but remained stable in the treatment group, indicating reduced oxidative stress. At 30 min post‐bypass, CK‐MB, thromboxane B2, and endothelin‐1 levels were significantly lower in group B, with MDA correlating with CK‐MB (*r* = 0.71, *p* = 0.0005). Endothelin‐1 negatively correlated with the 6‐keto‐prostaglandin F1α/thromboxane B2 ratio (*r* = −0.64, *p* = 0.0025) at 24 h post‐bypass. These findings suggest that SM reduces oxidative stress, mitigates myocardial damage, and improves the balance of vasoactive mediators after cardiopulmonary bypass, highlighting its potential as a cardioprotective therapy (Xia et al. 2003).

A similar conclusion was reached by Mu et al., who explored the cardioprotective effects of SM extract in an isoproterenol (ISO)‐induced acute myocardial ischemia (AMI) rat model. Rats were pretreated with SM extract (0.8, 0.9, and 1.8 g/kg/day), and their effects were assessed through histopathological analysis, cytokine levels, and untargeted metabolomics. LC–MS/MS analysis identified 25 major compounds in the SM extract, including salvianolic acid B, lithospermic acid, salvianolic acid A, and caffeic acid. Results indicated that SM extract markedly decreased myocardial infarct size, normalized electrocardiographic patterns, and repaired histopathological irregularities, lowered proinflammatory cytokines, and inhibited oxidative stress. Metabolomics analysis revealed 24 differential metabolites that returned to control levels post‐treatment, mainly linked to the metabolism of histidine, alanine, aspartate, glutamate, glycerophospholipids, glycine, serine, and threonine. Correlation analysis revealed that the heart‐protective effects of SM extract were associated with changes in endogenous metabolites. These results underscore SM extract as a potential therapeutic candidate for myocardial ischemia through anti‐inflammatory, antioxidative, and metabolic regulatory mechanisms (Mu et al. 2024).

## 7 Conclusion and Future Directions

Danshen ( *Salvia miltiorrhiza* ) shows cardioprotective potential, including antioxidant, anti‐inflammatory, vasodilatory, and anticoagulant effects. Its major bioactive classes—tanshinones and salvianolic acids—are associated with improved endothelial function, reduced oxidative stress, antithrombotic activity, and attenuation of cardiac remodeling. However, poor bioavailability, compositional variability, and regulatory barriers remain key limitations. Future research should focus on improving bioavailability using nanotechnology‐based delivery systems (e.g., liposomes, polymeric nanoparticles, SLNs, SMEDDS) and exploring alternative transdermal and injectable formulations. Mechanistic studies should further clarify roles in atherosclerosis, heart failure, and ischemic heart disease, while well‐designed clinical trials are needed to confirm safety, efficacy, and optimal dosing. Standardization using phytochemical fingerprinting and bioassay‐guided quality control will be essential for regulatory acceptance and clinical adoption. Potential herb–drug interactions with cardiovascular medications should also be systematically evaluated. Overall, integrating traditional knowledge with modern research may support Danshen as a complementary option in cardiovascular care, provided interdisciplinary work advances its safe clinical translation.

## Author Contributions

**Daniela Calina:** supervision, writing – review and editing, writing – original draft, investigation, visualization, validation, methodology, data curation. **William N. Setzer:** writing – review and editing, investigation, validation, methodology, data curation, supervision, visualization. **Preeti Patel:** writing – review and editing, writing – original draft, investigation, visualization, methodology, data curation. **Javad Sharifi‐Rad:** writing – original draft, writing – review and editing, visualization, validation, methodology, conceptualization, investigation, data curation, project administration, supervision. **Akash Vikal:** writing – review and editing, writing – original draft, investigation, visualization, methodology, data curation. **Rashmi Maurya:** writing – review and editing, writing – original draft, investigation, visualization, methodology, data curation. **Balak Das Kurmi:** writing – review and editing, writing – original draft, investigation, visualization, methodology, data curation.

## Funding

The authors have nothing to report.

## Conflicts of Interest

The authors declare no conflicts of interest.

## Data Availability

Data sharing not applicable to this article as no datasets were generated or analysed during the current study.
